# Detection of formal thought disorders in child and adolescent psychosis using machine learning and neuropsychometric data

**DOI:** 10.3389/fpsyt.2025.1550571

**Published:** 2025-03-17

**Authors:** Przemysław T. Zakowicz, Maksymilian A. Brzezicki, Charalampos Levidiotis, Sojeong Kim, Oskar Wejkuć, Zuzanna Wisniewska, Dominika Biernaczyk, Barbara Remberk

**Affiliations:** ^1^ Collegium Medicum, University of Zielona Gora, Zielona Góra, Poland; ^2^ Nuffield Department of Clinical Neurosciences, University of Oxford, Oxford, United Kingdom; ^3^ Department of Medicine, Poznan University of Medical Sciences, Poznan, Poland; ^4^ Department of Child and Adolescent Psychiatry, Institute of Psychiatry and Neurology, Warsaw, Poland

**Keywords:** child and adolescent psychosis, machine learning, cognitive psychiatry, executive deficits, formal thought disorder

## Abstract

**Introduction:**

Formal Thought Disorder (FTD) is a significant clinical feature of early-onset psychosis, often associated with poorer outcomes. Current diagnostic methods rely on clinical assessment, which can be subjective and time-consuming. This study aimed to investigate the potential of neuropsychological tests and machine learning to differentiate individuals with and without FTD.

**Methods:**

A cohort of 27 young people with early-onset psychosis was included. Participants underwent neuropsychological assessment using the Iowa Gambling Task (IGT) and Simple Reaction Time (SRT) tasks. A range of machine learning models (Logistic Regression (LR), Support Vector Machines (SVM), Random Forest (RF) and eXtreme Gradient Boosting (XGBoost)) were employed to classify participants into FTD-positive and FTD-negative groups based on these neuropsychological measures and their antipsychotic regimen (medication load in chlorpromazine equivalents).

**Results:**

The best performing machine learning model was LR with mean +/- standard deviation of cross validation Receiver Operating Characteristic Area Under Curve (ROC AUC) score of 0.850 (+/- 0.133), indicating moderate-to-good discriminatory performance. Key features contributing to the model’s accuracy included IGT card selections, SRT reaction time (most notably standard deviation) and chlorpromazine equivalent milligrams. The model correctly classified 24 out of 27 participants.

**Discussion:**

This study demonstrates the feasibility of using neuropsychological tests and machine learning to identify FTD in early-onset psychosis. Early identification of FTD may facilitate targeted interventions and improve clinical outcomes. Further research is needed to validate these findings in larger, more diverse populations and to explore the underlying neurocognitive mechanisms.

## 1 Introduction

Formal Thought Disorders (FTDs) comprise a range of complex psychiatric phenomena characterized by a disruption in the form (as opposed to the content) of thought processes (1). This distinguishes them from delusions, which are predominantly content based (2). Clinical examination of FTDs focus on the analysis of time-related parameters as well as qualitative (logical) structure of thoughts’ stream (3). Due to a broad spectrum of neuropathological changes affecting cognitive, linguistic, and emotional domains, FTDs can significantly impair a patient’s ability to communicate. This, in turn, compromises their quality of life and restricts social and occupational participation. The presence of FTDs is also a poor prognostic factor, heralding a limited response to therapeutics and a decreased rate of remission. FTDs are more prevalent in early-onset schizophrenia (54.5%) as compared to a late-onset schizophrenia (5.6%) and can even precede overt psychotic symptoms (4). Early-onset schizophrenia (EOS) spectrum is defined as the onset of psychotic symptoms below the 18 years of life (*early-onset)*, as well as below the 13 life year (*very-early onset*, VEOS). EOS populations links with different clinical picture, including: autism-spectrum features, psychoactive substances misuse, longer duration of untreated psychosis (DUP) and poorer prognosis. Thus, a timely and accurate identification of FTDs holds a significant value in child and adolescent psychiatric practice.

Quantifying formal thought disorders usually involves clinical assessment using standardized psychometric scales. Many such scales have been proposed. The most commonly used are the Thought Disorder Index (TDI) (5) and the Bizarre – Idiosyncratic Thinking scale (6). These are adequately reliable and feature a valid scoring system but tend to be time consuming. The well-established Thought, Language and Communication scale (TLC) (7) concisely encapsulates many aspects of thought disorders but does not include all of the FTD symptoms. This means that it may miss more subtle and early manifestations of FTD in children. The Thought and Language index (TLI) (8), while capable of detecting subtle disorders, fails to capture the more nuanced subjective elements, and its coding of verbal transcripts tends to be time-consuming as well. The Clinical language (CLANG) (9) scale investigates thought, as well as the speech quality (e.g. enunciation, impairments), but operates on a strict linguistic hypothesis, which means it may miss other domains of FTD presentation. Aiming towards a more holistic understanding of FTDs, the Formal Thought Disorder (FTD) scales (10) were introduced. These scales provide an explanation of thought disorders from the viewpoint of both patient and caregiver. However, they tend to lack an objective assessment of symptoms and may lead to misinterpretations due to the absence of clinical interviews complementing the assessment. The relatively newer Thought and Language Disorder (TALD) (11) scale offers a thorough and sensitive evaluation and encompasses the multidimensional range of FTD, but is less scientifically validated, and has a limited subjective evaluation (24 hrs prior to interview).

There is a clear need for a more objective and less interviewer-dependent way of assessing FTDs that could be more easily deployed in clinical practice. To that end, one interesting avenue of exploration presented itself in the cognitive domain of FTD (12). Positive FTD features such as circumstantiality, tangentiality, derailment or logorrhea have been linked to impairments of executive function (13). Similar executive deficits have been observed in negative FTD symptoms such as dysfunction of thought production (14). FTDs have also been associated with impairments in attention and working memory, as well as verbal and semantic fluency deficits (15). Despite a relative abundance of research in this area, results of some cognitive studies have been inconclusive (12, 16–18). The vast majority of the studied population were psychotic or manic adult patients (18), who may present with a different neurobiology of symptoms as compared with the child and adolescent population (19, 20). Furthermore, there is currently no research investigating whether executive dysfunction can be used to screen for the presence of FTDs in early psychosis patients. Since FTDs can be associated with poorer treatment responses, an early signal of the disease can be of clinical importance.

Machine learning approaches have emerged as powerful tools in psychiatric research, offering the potential to identify complex patterns in clinical, neurobiological, and behavioral data that may not be apparent through traditional statistical methods. The success of these applications, combined with the need for more objective and efficient diagnostic tools in early-onset psychosis, provides a strong rationale for investigating machine learning approaches in FTD detection.

The aim of the present study is to determine whether commonly used, rapid, and inexpensive executive function tests can be used to differentiate individuals with early-onset psychosis with an FTD element from those with similarly presenting early-onset psychosis, but without a discernible functional thought disorder. Our hypothesis is that individuals with aberrant neural networks will underperform in neuropsychometric testing due to the increased cognitive load of the underlying psychotic symptoms.

## 2 Methods and analysis

Measurements obtained for this paper originate from Comparison of Biomarkers in Schizophrenia and Bipolar Affective Disorder study, which was a case control study approved by the Regional Ethics Committee at Poznan University of Medical Sciences (reference number 1066/19), in line with European regulations. All participants and/or legal guardians received written information about the study prior to recruitment. Informed consent was taken by a Good Clinical Practice (GCP)-trained clinician member of the research team. The recruitment process was as follows. All patients admitted with psychotic symptoms to the Zabor Centre for Child and Adolescent Psychiatry (a tertiary referral hospital for western Poland) were offered participation in the study [for full protocol see (21)], and if eligible, were included in the present analysis. Diagnosis of FTD was made using Structured Clinical Interview for DSM Disorders (SCID-I) by a licensed child and adolescent psychiatrist and later confirmed by an independent board-certified specialist and principal provincial consultant for child and adolescent psychiatry. The FTD criteria used for the diagnosis were as per DSM-IV. The inclusion criteria were primary psychotic disorder and ability to perform cognitive task, as well as stabilization of medication. Exclusion criteria were presence of any acute medical or metabolic disorder, detection of alcohol or psychoactive substances during admission procedure or moderate-severe learning disability. Finally, in order to be eligible for inclusion, patients had to be clear of secondary psychosis, or other internal medicine diagnoses presenting with psychotic symptoms. All participants were tested in their on-treatment state, whereby stabilization and effective dosage of antipsychotic medication was achieved for at least 6 weeks. Pharmacological treatment is listed in **Table 1**.

**Table 1:** List of all antipsychotic medication used in treatment of participants included in the study, at the time of neuropsychometric evaluation.

Medication	Dosages	Chlorpromazine equivalent [mg]
Olanzapine	20mg	400
Aripiprazole	30mg	400
Aripiprazole + Fluoxetine	15mg + 20mg	200
Olanzapine + Chlorprothixene	10mg+45mg	302.5
Olanzapine + Aripiprazole + Fluoxetine	20mg+15mg+20mg	600
Olanzapine	20mg	400
Aripiprazole + Olanzapine + Valproic Acid	22.5mg+5mg+600mg	400
Olanzapine	10mg	200
Olanzapine + Valproic Acid	20mg+600mg	400
Aripiprazole + Olanzapine	15mg+5mg	300
Aripiprazole + Carbamazepine + Olanzapine	30mg +400mg +20mg	800
Aripiprazole + Valproic acid + Olanzapine	22.5mg+5mg+600mg	400
Aripiprazole + Sertraline	22.5mg+50mg	300
Aripiprazole	7.5mg	100
Escitalopram	20mg	0
Aripiprazole	30mg	400
Aripiprazole	30mg	400
Aripiprazole	30mg	400
Aripiprazole + Risperidone	30mg+1mg	500
Aripiprazole	22.5mg	300
Aripiprazole	30mg	400
None		0
Aripiprazole	30mg	400
Aripirazole	22.5mg	300
Risperidon + Fluvoxamine	1mg+100mg	100
Aripiprazole + Karbamazepine	30mg+300mg	400
None		0

Two tests were used in this study: the Iowa Gambling Task (IGT) and the Simple Response Time (SRT), which were chosen for their ubiquitous use in assessment of executive function. IGT was administered by Psychological Experiment Building Language (PEBL) software using standard protocol (22). SRT was delivered by the same software, with the task featuring an instruction to press the left mouse button upon presentation of a visual stimulus on the screen (a letter X). The features for these tasks were extracted as follows. For IGT, the number of advantageous cards (sum of, out of 100) and disadvantageous cards (sum of, out of 100) were used. For SRT, the mean, standard deviation was used for the 4 x 50 trials, with breaks in between. Minimum and maximum reaction times were also used.

A range of machine learning models were used for classification, namely LR, SVM, RF, and XGBoost. The choice of models was based on their versatility in handling small size samples. Feature scaling was performed using standardization to ensure comparable feature ranges. To optimize model performance and prevent overfitting, a standardized sklearn library pipeline was used, incorporating standardization (Standard Scaler) and model fitting. Hyperparameter tuning was conducted using a grid search with the parameters shown in **Table 2**.

**Table 2:** Hyperparameter tuning of the machine learning models.

Model	Parameters
Linear regression	‘classifier:penalty’: [‘l1’, ‘l2’], ‘classifier:C’: np.logspace(-4, 4, 20), ‘classifier:solver’: [‘liblinear’, ‘saga’]
Support Vector Machines	‘classifier:C’: [0.1, 1, 10], ‘classifier:kernel’: [‘rbf’, ‘linear’, ‘poly’, ‘sigmoid’], ‘classifier:gamma’: [‘scale’, ‘auto’] + list(np.logspace(-4, 4, 20)),
Random Forest	‘classifier:n_estimators’: [50, 100, 200] ‘classifier:max_depth’: [None, 10, 20] ‘classifier:min_samples_split’: [2, 5, 10] ‘classifier:min_samples_leaf’: [1, 2, 4] ‘classifier:max_features’: [‘sqrt’, ‘log2’, None]
XGBoost	‘classifier:n_estimators’: [50, 100, 200], ‘classifier:max_depth’: [3, 6, 9], ‘classifier:learning_rate’: [0.01, 0.1, 0.3], ‘classifier:gamma’: [0, 0.1, 0.2], ‘classifier:subsample’: [0.6, 0.8, 1.0], ‘classifier:colsample_bytree’: [0.6, 0.8, 1.0], ‘classifier:reg_alpha’: [0, 0.1, 1], ‘classifier:reg_lambda’: [0, 0.1, 1]

Nested cross-validation was implemented to provide a robust estimate of model performance. The outer loop employed a stratified five-fold cross-validation to partition the data into training and testing sets. For each training set, an inner five-fold cross-validation was performed to select the optimal hyperparameters based on the area under the receiver operating characteristic curve (AUC). Model performance was evaluated using a range of metrics, including accuracy, precision, recall, F1-score, and AUC. Additionally, a confusion matrix was generated to evaluate the model’s classification accuracy. Our analysis uses the default 0.5 probability threshold for class separation when calculating accuracy, precision, recall and F1-score; this threshold was not explicitly changed or optimized within training process.

### 2.1 Results

Out of N = 31 patients experiencing psychotic episode, N = 27 (N = 11 FTD negative; N = 16 FTD positive, as diagnosed by SCID-I) were well enough to complete neuropsychometric tests. Demographic and clinical information about this sample is presented in **Table 3**.

**Table 3:** Demographic data of studied population.

	FTD +	FTD -	P (test)
N	16	11	
Gender assigned at birth [M: F]	9:7	8:3	0.39 (chi-squared)
Age [mean years ± std]	14.4 ± 1.8	14.6 ± 1.7	0.78 (t-test)
Family history of psychosis [Y: N]	15:1	8:3	0.13 (chi-squared)
Family history of other psychiatric disorders [Y: N]	9:7	8:3	0.39 (chi-squared)
PANSS at baseline	115.8 ± 15.2	101.5 ± 21.7	0.06 (t-test)
PANSS at 6-8 weeks	53.7 ± 14.3	61.1 ± 29.9	0.42 (t-test)
Years of education	7.4 ± 1.8	7.6 ± 1.7	0.78 (t-test)
Learning disability category	Cat. 0 = 16	Cat. 0 = 10 Cat. 1 = 1	0.85 (chi-squared)

FTD, Formal thought disorder; STD, Standard deviation; PANSS, Positive and Negative Syndrome Scale. Learning disability as per psychologists’ assessment (neuropsychometry + gestalt evaluation) with the following categories: 0, No intellectual disability; 1, Mild; 2, Moderate; 3, Severe; 4, Profound. s

**Figure 1:**
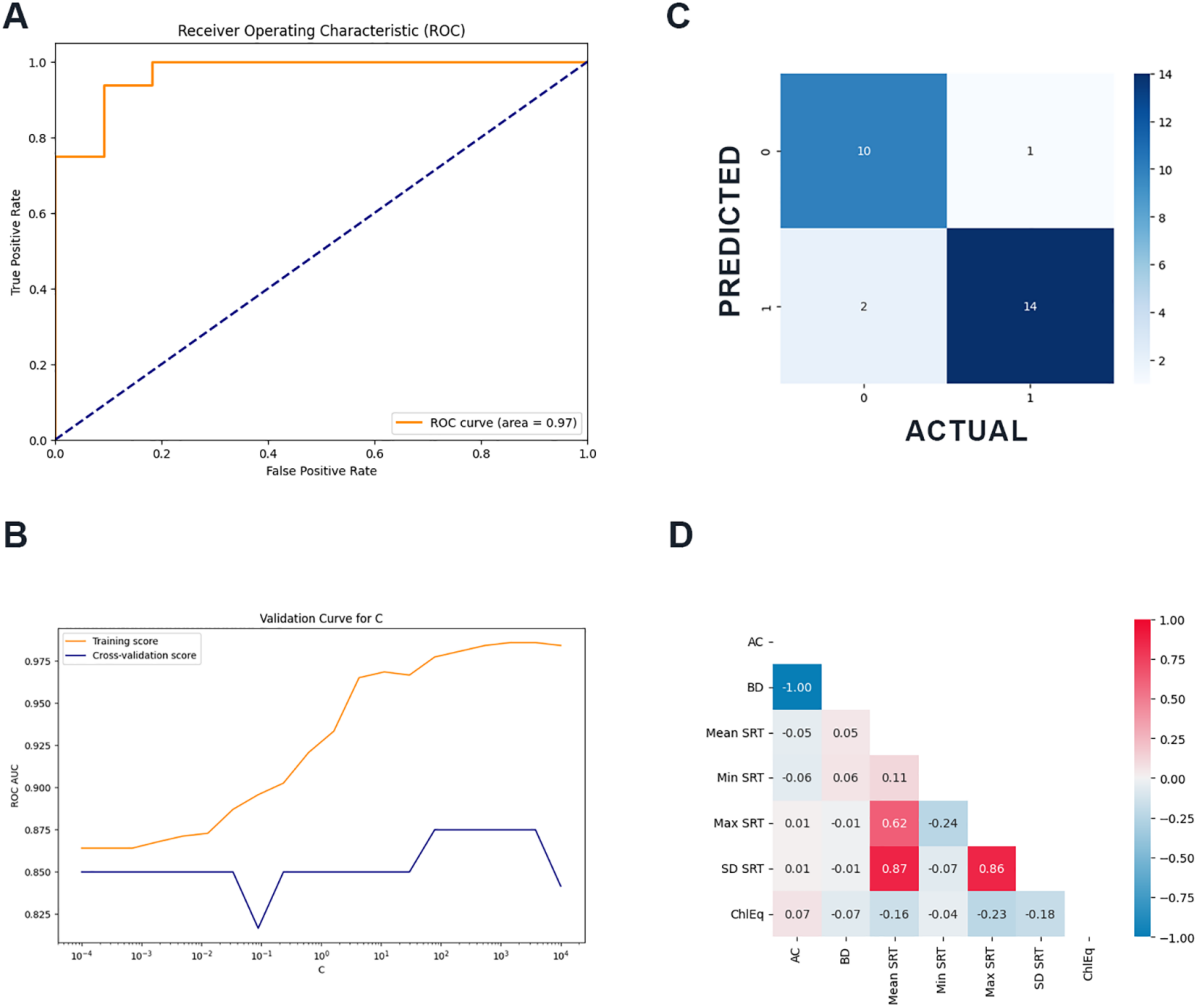
**(A)** Receiver operating characteristic curve of the winning model for detecting formal thought disorder in child and adolescent patients experiencing psychosis **(B)** Validation curve for the model **(C)** confusion matrix showing overall classification performance of the model **(D)** Correlation matrix of the features included in the model.

Nested cross-validation results for all models are presented in **Table 4**. The winning model was Logistic Regression (mean +/- standard deviation): Accuracy: 0.673 (+/- 0.213), Precision: 0.750 (+/- 0.224), Recall: 0.750 (+/- 0.247), ROC AUC: 0.850 (+/- 0.133), F1: 0.723 (+/- 0.195). Validation curve for C can be found in **Figure 1B**. The final feature coefficients (relative contributions) to the winning model were advantageous cards: -0.299, disadvantages cards: 1.645, mean SRT: 2.592, minimum SRT: 2.428, maximum SRT: 3.617, standard deviation of SRT: -6.360, and chlorpromazine equivalent in milligrams: 2.187. The correlation matrix revealed an expected perfect (1) correlation of IGT advantageous and disadvantageous card selections and an equally anticipated high (0.86) correlation between maximum SRT reaction time and standard deviation of the SRT reaction time. The remainder of features were not significantly correlated (**Figure 1D**).

**Table 4:** Model performance evaluation.

Model	Accuracy	Precision	Recall	Roc_auc	F1
Linear Regression	0.673 (+/- 0.213)	0.750 (+/- 0.224)	0.750 (+/- 0.247)	0.850 (+/- 0.133)	0.723 (+/- 0.195)
Support Vector Machines	0.740 (+/- 0.090)	0.783 (+/- 0.113)	0.817 (+/- 0.153)	0.781 (+/- 0.081)	0.786 (+/- 0.072)
Random Forest	0.700 (+/- 0.099)	0.717 (+/- 0.041)	0.817 (+/- 0.153)	0.713 (+/- 0.149)	0.760 (+/- 0.085)
eXtreme Gradient Boosting	0.660 (+/- 0.090)	0.673 (+/- 0.067)	0.883 (+/- 0.145)	0.753 (+/- 0.163)	0.755 (+/- 0.061)

Results shown are mean (+/- standard deviation).

The overall ROC AUC for the best model was 0.972 (**Figure 1A**). The confusion matrix provides a detailed overview of the winning model’s classification performance. As shown in **Figure 1C**, based on neuropsychometry data alone, the model was able to correctly classify 10 participants without FTD and 14 with FTD. One participant was incorrectly classified as having FTD, whilst two were predicted as not having FTD diagnosis despite being confirmed by ground truth psychiatric assessment. Overall, the model demonstrated moderate-to-good performance in assigning participants to both classes.

## 3 Discussion

This study demonstrated the potential of neuropsychological testing, specifically the IGT and SRT, combined with machine learning, to differentiate individuals with and without formal thought disorders within a cohort of children and adolescent experiencing psychotic episodes. A logistic regression model, optimized through nested cross-validation, achieved moderate-to-good discriminatory performance. The model’s classification accuracy revealed correct identification of all but three participants. Feature analysis highlighted the significant contributions of the minimum and standard deviation SRT reaction time, and IGT advantageous and disadvantageous card selections to the model’s predictive capacity. No differences were observed in overall disease severity as assessed by PANSS, at baseline and after treatment, which may support the added clinical value of neuropsychometric testing in this population. These findings suggest that distinct patterns of decision-making and reaction time variability, as measured by the IGT and SRT, respectively, may serve as valuable indicators in the diagnostic process of FTD in individuals presenting with psychosis.

Feature importance analysis yield further insights into the utility of neuropsychometric testing, as response time measurement enables real-time and independent clinical insight into executive processing. SRT reflects the quality of processing speed in cortico-subcortical loops, crucial for higher cognitive functions like thinking operations. According to dysexecutive theory of FTDs, thinking disorders may be the effect of general processing speed disorder. Among general population the software functions like thinking and language networks operate on healthy background (hardware) of brain cybernetics. Regarding psychotic patients, impaired linguistic networks operations and decision making may be the effect of brain disconnection and cognitive load due to concurring processes, like aberrant salience phenomena. Hence the minimal and standard deviation of SRT may be an important proxy feature for the background functioning of the brain hardware.

To our knowledge this is the first study specifically investigating the use of SRT and IGT cognitive modalities in child and adolescent psychotic population. The executive function (EF) impairments demonstrated through these tests add to the body of evidence suggesting that goal-directed behavior deficits are prevalent in children and adolescents at risk for psychosis, as well as those diagnosed with schizophrenia spectrum disorders. A study involving 100 genetically high-risk children and adolescents identified four neurocognitive clusters based on EF performance alone, highlighting significant impairments self-control and decision-making capabilities (23). Another study found that poor “hot” executive functions, such as decision-making under uncertainty, were significantly associated with psychotic symptoms in a cohort of 156 young patients, suggesting a specific vulnerability in spatial working memory and Cambridge Gambling Task (24). Research on adolescents with antipsychotic-treated schizophrenia showed that these individuals had higher scores on the Behavior Rating Inventory of Executive Function, indicating notable EF impairments compared to healthy controls (25). Additionally, a study of adolescents with schizophrenia spectrum disorders revealed that executive function was among the most impaired cognitive domains, paralleling findings in adult populations (26). EF impairments were also predictive of persistent semantic and language production impairments in first episode psychosis population.

The results of our experiment can be corroborated by measurements obtained in adult psychotic population. One study revealed a moderate (R2 = -0.48) correlation of Wechsler Adult Intelligence Scale processing speed and FTD symptoms in adults fulfilling the DSM IV-TR criteria for schizophrenia disorder (27). Similar observations (R2 = -0.54) were made for association of communication disturbance index (a measure of FTD) and processing speed in an arrow task, whereby patients with primary psychotic disorder needed to react to the arrow pointing right or left and choose the correct or opposite direction on the basis of the color of the cue (28). FTD symptoms also seem to intensify when participants are asked to perform cognitive tasks, which may explain why their real-life functioning is often impaired (29).

The connection between FTD and EF deficits observed in our population have a strong neurobiological basis, which may be understood in three dimensions: the anatomical impact, the neurotransmitter system dysregulation, and the genetic background (2). One of the major findings in structural MRI in schizophrenic patients is the reduction of cortical volumes in the left superior temporal gyrus, middle temporal gyrus, and the frontal operculum (30). The affected areas are within the Wernicke’s and Broca’s areas, therefore, possibly affecting the neurodevelopment of language and speech (31). Findings from Kircher et al., also suggest that dysfunctional glutamatergic receptors caused by reduced synapses in the superior temporal gyrus are the main drive for developing positive FTD. Moreover, dopaminergic hyperactivity and hyper-priming has shown to affect both glutamatergic excitatory and GABAergic inhibitory networks, causing positive FTD in schizophrenia (2). The pathophysiology of psychotic state in schizophrenia involves both dopaminergic dysregulations in aberrant salience and neural network synaptic plasticity disruption linking with loosening of associations. Neurobiological processes, underlying the psychotic pathology of the brain, show impaired positive reinforcement cybernetic loop between dopaminergic outbursts and synaptic disorganization, enhancing each other. Lastly, twin studies and adoption studies have shown a positive correlation between FTD and heritability, suggesting genetic risk of developing FTD (32, 33). It may be hypothesized that the poor treatment outcomes for FTD population stem from a lack of adequate focus on these neurobiological pathways in conventional therapeutic options for child and adolescent psychotic population. The pharmacological options deployed in FTD cases often target acute positive FTD, utilizing neuroleptic action and providing the stabilized dopaminergic pathway without any effects on cortical plasticity mechanisms. There are a few studies supporting the effects of cognitive-behavioral therapy (CBT) on FTD (34). However, no specific psychotherapy has so far been established.

The implementation of machine learning-based FTD detection in clinical settings appears feasible, given the relatively straightforward administration of IGT and SRT tasks requiring only standard computing equipment and a few minutes of testing time. The model’s moderate-to-good discriminatory performance suggests utility as a screening tool to complement traditional clinical assessment, potentially improving early detection of FTD in resource-limited settings. Caution must be applied, however, as all small number studies have inherent limitations regarding their external validity in real-life settings. Integration into existing workflows could thus follow a two-stage approach, where positive machine learning-based screening results trigger comprehensive clinical evaluation, while regular administration could facilitate longitudinal monitoring of cognitive function and treatment response.

The study is not without limitations. First, we only used two relatively simple tasks, producing features with very limited granularity and cognitive domain precision. This was done to maximize participation and enhance clinical adaptation, which is easier with simpler and less time-consuming tasks. Not all participants were correctly classified by the model. Therefore, *post-hoc* analysis of medical notes was conducted to look for any clinicodemographic differences that may explain these discrepancies. In one false-negative case, the treating clinicians recorded a rapid onset of symptoms, with a short duration of untreated psychosis. It may be hypothesized that an early stabilization of the disease may have spared cognitive function in this case. Conversely, in two false-positive cases, the treatment duration was relatively long, with repeated extensive stay hospitalizations and exacerbations of the disease. This may have caused greater cognitive decline despite lack of the FTD element. Further research on a larger population is clearly warranted to establish whether there is any significant link between FTD and clinicodemographic features such as disease duration, number of hospitalizations, specific drug agents, or treatment intensity.

## Data Availability

The original contributions presented in the study are included in the article/supplementary material. Further inquiries can be directed to the corresponding author.
